# Two cases of *Streptococcus equi* subspecies *zooepidemicus* bacteremia diagnosed in a French hospital within a short space of time

**DOI:** 10.1007/s10096-026-05410-x

**Published:** 2026-01-24

**Authors:** Guillaume Morin, Laura Chaufour, Marion Lacasse, Claudia Carvalho Schneider, Marie-Frédérique Lartigue, Coralie Lemaire

**Affiliations:** 1 Service de Bactériologie-Virologie-Hygiène Hospitalière, CHRU de Tours, Tours, France; 2 Université de Tours, INRAE, Infectiologie et Santé Publique, BRMF, Tours, France; 3 Service de Maladies Infectieuses et Tropicales, CHRU de Tours, Tours, France

**Keywords:** S. equi ssp. zooepidemicus, Bacteraemia, Cheese

## Abstract

*Streptococcus equi* subspecies *zooepidemicus* is an opportunistic pathogen. Human infections are rare and often linked to close contact with horses. Consumption of raw milk cheese has also been incriminated. We present two cases of *S. equi* ssp. *zooepidemicus* bacteremia diagnosed within a short space of time in the same hospital.

## Introduction

*Streptococcus equi* subspecies *zooepidemicus* is a Gram-positive ß-hemolytic coccus, typically arranged in pairs or chains, which produces the Lancefield Group C antigen. It is part of the commensal microbiota of the skin and mucous membranes of horses (respiratory, genital, etc.), but can commonly become pathogenic and be responsible, for example, for pneumonia or neonatal infections in these animals [1]. It is involved in infections in other mammals such as pigs [2], dogs [3], cattle [4], cats [5], sheep [6] but also, and more rarely, in humans [7]. In the latter, it has been described in cases of bacteremia [8], meningitis [9, 10], arthritis [11], aneurysm [12], etc. Human contamination often occurs through close contact with carrier or infected animals, particularly horses, but transmission has also been reported with cats and dogs [13, 14]. It has also been described through consumption of raw milk cheese (probably from cows with *S. equi* ssp. *zooepidemicus* mastitis) [15, 16] or raw pork products [17] and can lead to glomerulonephritis [18, 19].

However, this bacterium remains rarely isolated in human clinical microbiology laboratories. Here we present two cases of bacteremia caused by *S. equi* ssp. *zooepidemicus*, isolated within a short period of time in the bacteriology laboratory of Tours University Hospital.

## Cases presentation

### Case 1

An 89-year-old man was admitted at the Tours University Hospital (France) on January 25, 2024 for an altered general condition and numerous falls without loss of consciousness. He was a retired sales agent who lived in the city (Tours), did not drink alcohol or smoke, and reported no contact with horses but ate some unpasteurized raw sheep’s milk cheese few days before.

On arrival, the patient presented with infectious, neurological, and respiratory signs, and a painful erythematosus left mandibular mass was visible (Table 1). He did not have fever at the Emergency Room, but it appeared throughout his stay. A body Computed Tomography (CT) Scan showed submaxillary parotitis with infiltration and mass effect on the respiratory tract. A large thyroid goiter, pleural effusion and a false aneurysm of the descending aorta opposite the esophageal hiatus were also detected. A significant inflammatory syndrome was observed, with elevated C-reactive protein (175 mg/L) and hyperleukocytosis (30 G/L).

**Table 1:** Description of the clinical and biological features of the two cases

	Case 1	Case 2
Gender/age	89-year-old man	75-year-old man
Comorbidities	Heart disease, high blood pressure, prostate cancer (in remission)	Diabetes, dyslipidemia, high blood pressure, sleep apnea syndrome, chronic kidney disease, chronic arthritis, atrial fibrillation, aortic bioprosthesis, prostate cancer
Clinical signs	Altered general condition with numerous falls, submaxillary parotitis, large thyroid goiter, pleural effusion and a false aneurysm of the descending aorta	Asthenia, functional urinary signs, arthralgia and fever
Diagnosis	Aortitis caused by *S. equi* ssp. z*ooepidemicus* and *S. aureus* (associated bacteremia)	*S. equi* ssp. z*ooepidemicus* bacteremia complicated by knee prosthesis infection
Treatment	Amoxicillin + gentamicin IV (1 day)/daptomycin IV (1 day)/cefazolin IV (7 days)/trimethoprim/sulfamethoxazole *per os* (1 day)/amoxicillin + clindamycin (for a total of 6 weeks of treatment)	Ceftriaxone IV (1 day)/amoxicillin IV (3 weeks)/amoxicillin *per os* (for a total of 3 months of treatment)
Outcomes	Favorable progress, no chronic infection (July 2025)	Favorable progress, no residual pain in his left knee (November 2025)

**Fig. 1:**
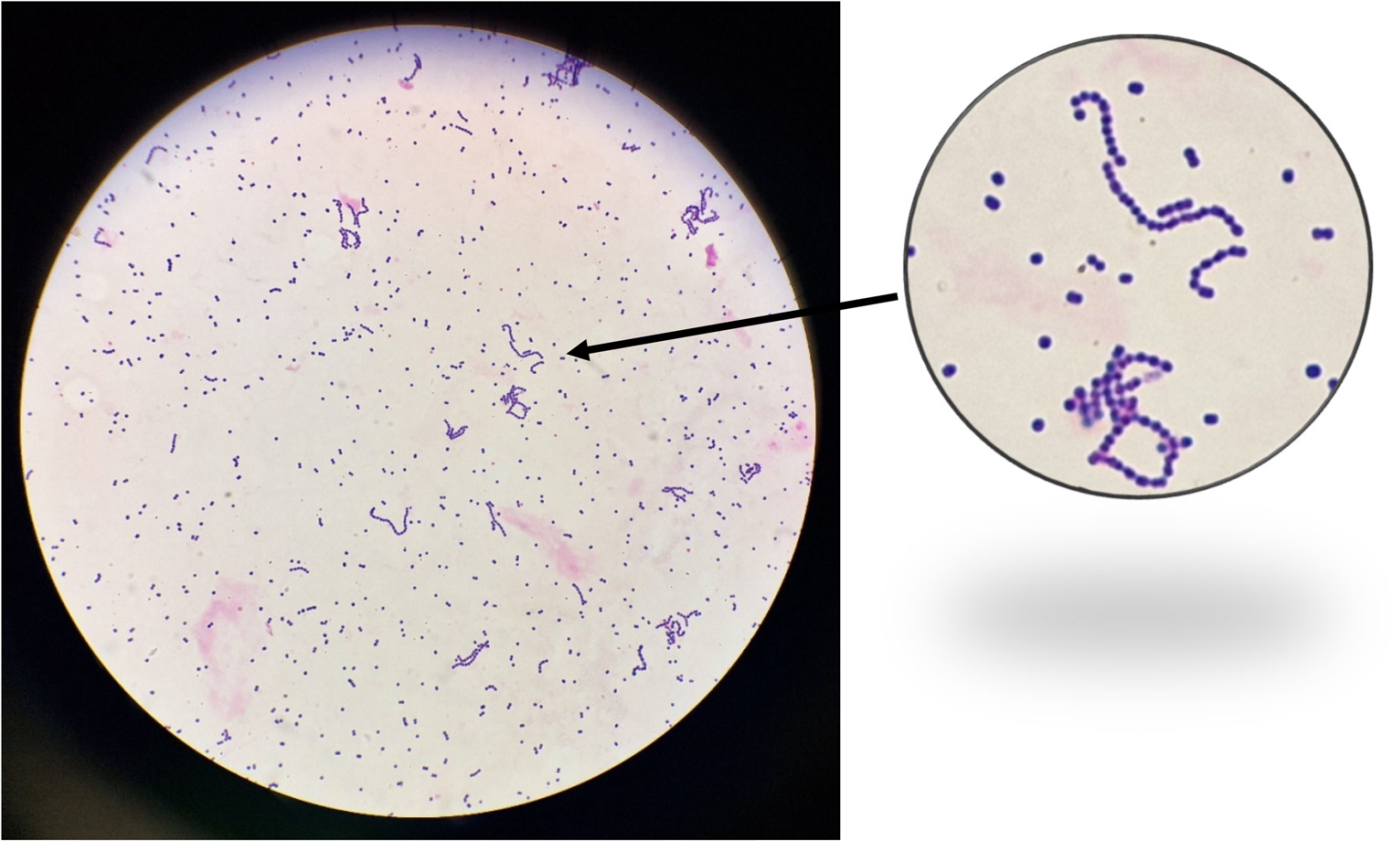
Gram staining of blood cultures, showing Gram-positive diplococci or cocci in short chain (GX1000)

**Fig. 2:**
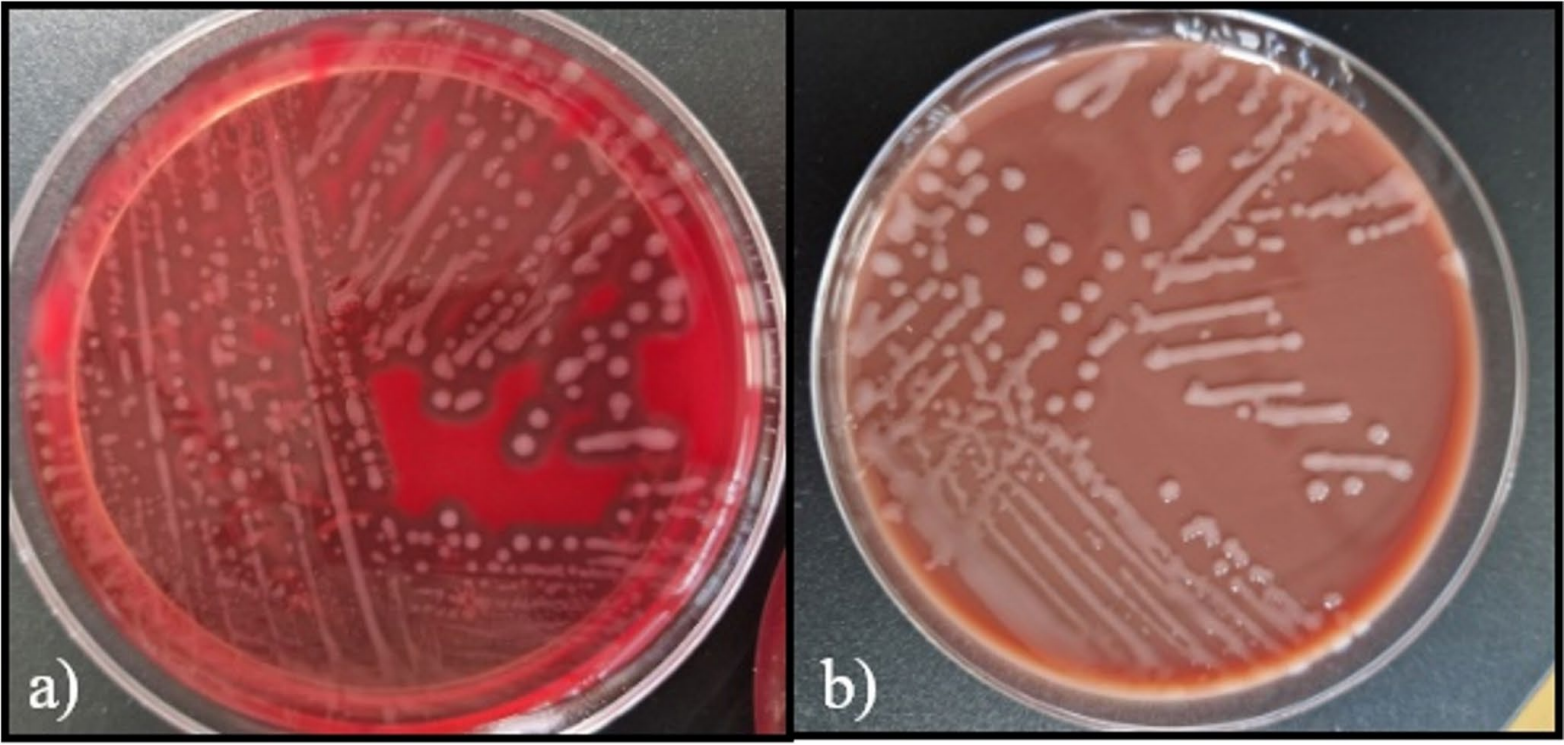
Culture of *S. equi* ssp. *zooepidemicus* (**a**) on blood agar (**b**) on chocolate agar, after incubation for 24 h at 37 °C, respectively in aerobic and in 5% CO2 atmosphere

The next day, two pairs of blood cultures became positive. Gram-positive cocci in chains were observed in both vials (aerobic and anaerobic) of both sets (Fig. 1). On blood agar, β-hemolytic and mucous colonies grew (Fig. 2.a and b). Matrix-Assisted Laser Desorption/Ionization-Time of Flight mass spectrometry (MALDI-TOF MS, Bruker Daltonics GmbH, Bremen, Germany) identified *S. equi* ssp. *zooepidemicus* (MBT IVD reference, version 2023, score 2.01). Sequencing of the 16 S ribosomal DNA (16 S rDNA) [20] and *sodA* [21] genes confirmed this identification. The strain was susceptible to beta-lactams (Table 2), according to French guidelines, Antibiotic Susceptibility Committee of the French Society of Microbiology 2022 (CA-SFM 2022, https://www.sfm-microbiologie.org). Amoxicillin (6 g twice a day) and gentamicin (1 injection of 3 mg/kg) were introduced intravenously. A stent was placed to treat the aneurysm. *Staphylococcus aureus* was also identified in culture on both vials of the second set of blood cultures (not on the first set), prompting a change in antibiotic therapy to daptomycin intravenous (IV) (750 mg a day). To resume, there was only *S. equi* ssp. *zooepidemicus* in the two vials of the first set and *S. equi* ssp. *zooepidemicus* and *S. aureus* in the two vials of the second set (no clusters of cocci were seen on direct examination). As the *S. aureus* was sensitive to methicillin, the following day the daptomycin was replaced by cefazolin IV (2 g twice a day). After 7 days of cefazolin, trimethoprim/sulfamethoxazole *per os* was prescribed to treat a potential infectious aortitis. Due to deteriorating renal function, antibiotic therapy was replaced the next day by amoxicillin and clindamycin for six weeks. The patient was transferred to continue his care in a Rehabilitation department. Unpasteurized raw sheep’s milk cheese eaten by the patient may be suspected of being the cause of the patient’s contamination (*S. equi* ssp. *zooepidemicus)*, because no contact with animals has been reported by the patient, but unfortunately it was not possible to test the food to confirm our hypothesis. *S. aureus* can cause gastrointestinal infections following the consumption of cheese, but does not appear to cause bacteremia following food ingestion. It was only found in the second pair of blood cultures, but no entry point was found, particularly no skin lesion so the entry point is still unknown. Today, the patient is doing well, has gained weight and regained his autonomy at home. His endoprosthesis placed to treat the aneurysm presents no signs of infection.

**Table 2:** Antibiotics susceptibility from both *S. equi* ssp. *Zooepidemicus* strains

Antibiotics	S. equi ssp. Case 1	zooepidemicus strains Case 2
Penicillin G	Susceptible	Susceptible
Amoxicillin	Susceptible	Susceptible
Cefotaxime	Susceptible	Susceptible
Ceftriaxone	Susceptible	Susceptible
Gentamicin	High resistance level	Low resistance level
Clindamycin	Resistant	Susceptible
Pristinamycin	Susceptible	Susceptible
Erythromycin	Resistant	Susceptible
Levofloxacin	Resistant	Susceptible, increased exposure
Vancomycin	Susceptible	Susceptible
Teicoplanin	Susceptible	Susceptible
Rifampicin	Susceptible	Susceptible
Tetracycline	Resistant	Resistant
Thrimetoprim/sulfamethoxazol	Susceptible	Susceptible

## Case 2

A 75-year-old man was admitted to the Tours University Hospital (France) on February 1 st, 2024 for asthenia, functional urinary signs, and arthralgia (Table 1). Notably, he had a prosthetic aortic valve and a left knee prothesis. He is a retired anaesthesiologist and is self-sufficient at home. He does not drink alcohol, does not smoke, reports no contact with horses but ate Comté cheese rind (cow’s milk cheese) in the Jura region the previous week.

The patient had a body CT Scan at Hospital which revealed a bladder globe and a faecaloma. An antibiotic therapy with Ceftriaxone (1 g a day) was introduced. He was transferred to the Intensive Care Unit for a sepsis. An infectious profile of the patient, including blood culture, was initially performed. A significant inflammatory syndrome was observed with elevated C-reactive protein (398 mg/L) but no hyperleukocytosis. Blood cultures finally became positive (aerobic and anaerobic vials for each of the two sets) and direct examination with Gram staining showed short chains of Gram-positive cocci. On blood agar, β-hemolytic and mucous colonies grew and MALDI-TOF MS identified *S. equi* ssp. *zooepidemicus* (MBT IVD reference, version 2023, score 2.03). Sequencing of the 16 S rDNA and *sodA* genes confirmed this identification. The strain was susceptible to all tested antibiotics except tetracycline (Table 2), according to French guidelines (CA-SFM 2022). As the patient had a prosthetic aortic valve and streptococci are strongly implicated in endocarditis, a transthoracic echocardiography and a transesophageal echocardiography were performed. There was no evidence of endocarditis and spinal cord Magnetic Resonance Imaging (MRI) showed no evidence of spondylodiscitis. The antibiotic therapy was switched to amoxicillin IV (12 g a day) for two weeks. Five days later, the patient presented with inflammation and pain in his left knee, which prompted a joint puncture. The culture remained sterile but a *16 S rDNA* PCR was performed as part of antibiotic therapy before joint puncture. This analysis highlighted the presence of *S. equi* ssp. *zooepidemicus* in the joint liquid. Thus, the moving parts of this prothesis were replaced and a synovectomy was performed. Deterioration in the patient’s renal function led to a reduction in amoxicillin doses (4 g twice daily) for one week. A follow-up transthoracic echocardiogram was performed but did not reveal endocarditis. Antibiotic treatment was changed to oral amoxicillin (6 g daily) for a total period of three months. In total, the patient received three weeks of intravenous antibiotic treatment, followed by oral antibiotic treatment for three months. At the beginning of March, the patient was transferred to continue his care in a rehabilitation department. The rind of the cow’s cheese may therefore be suspected of causing of the bacteremia, as the patient reported no contact with farm animals like sheep or cows, but again, it was not possible to test the food to confirm our hypothesis.

## Conclusion

Two cases of *S. equi* ssp. *zooepidemicus* bacteremia were diagnosed at Tours Hospital (France) within two weeks. As this bacterium is rarely found, a common origin was initially suspected. After a complete interview of the patients, no common origin could be identified and the suspicion was quickly dispelled since the antibiograms are different. Most of the time, this bacterium is found following close contact with animals, particularly horses which carry it. Another means of contamination is the consumption of unpasteurized dairy products or raw pork, for example, contaminated by the bacterium. In both cases, the patients reported having consumed unpasteurized cheese, one from a sheep and the other from a cow. These cheeses may have been the source of the bacteremia observed, and the close timing of the two diagnoses is probably due to chance. Despite the few cases of human infections, *S. equi* ssp. *zooepidemicus* is well described and characterized in veterinary medicine. Veterinary microbiology enables human medicine to better understand the pathogenicity of zoonotic pathogens. Recent studies have shown that the carriage of *S. equi* ssp. *zooepidemicus* in animals may be more significant in certain regions in certain animal species, and analyses have been performed on these carrier strains. The results show significant diversity between strains, with varied sequence types, some of which possess virulence genes similar to those found in *Streptococcus pyogenes* (a virulent pathogen involved in human disease) [22]. In clustered cases, such as that involving donkeys, a particular sequence type has been identified [23]. Analysis of these strains revealed the presence of virulence genes that could explain the pathogenicity of these strains.

In clustered cases, it is therefore interesting to study the strain involved in order to detect the presence of virulence genes and better understand those involved in human pathology. Unfortunately, in our cases, the strains could not be analysed, and the potential source of contamination could not be confirmed. The suspect cheeses did not appear to have any common features (place of production, storage). It is therefore necessary to remain vigilant with regard to human infections with *S. equi* ssp. *zooepidemicus* and to objectively identify the sources of contamination in order to highlight any increase in the incidence of these infections. These cases alone do not allow to draw any conclusions. Cheese is indeed a potential source of contamination, but there are others, such as contact with animals. The patients report no contact with animals and do not have pets, but cases have been described following contact with domestic dogs and cats and cannot be ruled out based solely on patient interviews.

## Data Availability

No datasets were generated or analysed during the current study.
